# Association of the Built Environment With Childhood Psychosocial Stress

**DOI:** 10.1001/jamanetworkopen.2020.17634

**Published:** 2020-10-21

**Authors:** Meredith Franklin, Xiaozhe Yin, Rob McConnell, Scott Fruin

**Affiliations:** 1 Department of Preventive Medicine, Keck School of Medicine, University of Southern California, Los Angeles

## Abstract

**Importance:**

Emerging research suggests that factors associated with the built environment, including artificial light, air pollution, and noise, may adversely affect children’s mental health, while living near green space may reduce stress. Little is known about the combined roles of these factors on children’s stress.

**Objective:**

To investigate associations between components of the built environment with personal and home characteristics in a large cohort of children who were assessed for perceived stress.

**Design, Setting, and Participants:**

In this cohort study, a total of 2290 Southern California Children’s Health Study participants residing in 8 densely populated urban communities responded to detailed questionnaires. Exposures of artificial light at night (ALAN) derived from satellite observations, near-roadway air pollution (NRP) determined from a dispersion model, noise estimated from the US Traffic Noise Model, and green space from satellite observations of the enhanced vegetation index were linked to each participant’s geocoded residence.

**Main Outcomes and Measures:**

Children’s stress was assessed at ages 13 to 14 years and 15 to 16 years using the 4-item Perceived Stress Scale (PSS-4), scaled from 0 to 16, with higher scores indicating greater perceived stress. Measurements were conducted in 2010 and 2012, and data were analyzed from February 6 to August 24, 2019. Multivariate mixed-effects models were used to examine multiple exposures; modification and mediation analyses were also conducted.

**Results:**

Among the 2290 children in this study, 1149 were girls (50%); mean (SD) age was 13.5 (0.6) years. Girls had significantly higher perceived stress measured by PSS-4 (mean [SD] score, 5.7 [3.4]) than boys (4.9 [3.2]). With increasing age (from 13.5 [0.6] to 15.3 [0.6] years), the mean PSS-4 score rose from 5.6 (3.3) to 6.0 (3.4) in girls but decreased for boys from 5.0 (3.2) to 4.7 (3.1). Multivariate mixed-effects models examining multiple exposures indicated that exposure to secondhand smoke in the home was associated with a 0.85 (95% CI, 0.46-1.24) increase in the PSS-4 score. Of the factors related to the physical environment, an interquartile range (IQR) increase in ALAN was associated with a 0.57 (95% CI, 0.05-1.09) unit increase in the PSS-4 score together with a 0.16 score increase per IQR increase of near-roadway air pollution (95% CI, 0.02-0.30) and a −0.24 score decrease per IQR increase of the enhanced vegetation index (95% CI, −0.45 to −0.04). Income modified the ALAN effect size estimate; participants in households earning less than $48 000 per year had significantly greater stress per IQR increase in ALAN. Sleep duration partially mediated the associations between stress and both enhanced vegetation index (17%) and ALAN (18%).

**Conclusions and Relevance:**

In this cohort study, children’s exposure to smoke at home in addition to residential exposure to ALAN and near-roadway air pollution were associated with increased perceived stress among young adolescent children. These associations appeared to be partially mitigated by more residential green space. The findings may support the promotion of increased residential green spaces to reduce pollution associated with the built environment, with possible mental health benefits for children.

## Key Points

**Question:**

Are environmental exposures, including artificial light at night (ALAN), near-roadway air pollution, noise, green space, and secondhand smoke, associated with childhood psychosocial stress?

**Findings:**

In this cohort study of 2290 children, perceived stress was significantly associated with increased ALAN, near-roadway air pollution, and exposure to secondhand smoke at home, while residential green space appeared to partially mitigate the associations with these factors. Income appeared to modify the ALAN effect size estimate and sleep duration to partially mediate the associations between stress and both green space and ALAN.

**Meaning:**

Reducing artificial light and air pollution exposures by increasing green spaces may be associated with improvements in children’s mental health.

## Teaser

This cohort study examines stress levels in children based on environment factors, including artificial light at night, near-roadway air pollution, noise, green space, and secondhand smoke.

## Introduction

According to the last US census, urban areas account for more than 80% of the US population, and the growth rate of the urban population outpaced that of the rural population.^1^ Urban environmental stressors, including artificial light, air pollution, and noise, are ubiquitous in areas of high population density, and there is a growing body of research linking these aspects of the urban landscape to mental health.^2,3^ Combined with home and neighborhood factors, including exposure to secondhand smoke, family stress, crowding, and low household income, these stressors may affect children’s health through key regulatory systems in the body, such as cortisol levels.^4^ Increased psychosocial stress responses occurring in critical stages of childhood development can have measurable and lasting health effects.^5,6,7,8,9^

Artificial light at night (ALAN) is considered one of the most pervasive environmental pollutants of the built environment, and its prevalence has been shown to have substantial social, behavioral, and health consequences.^10^ It is well established that ALAN causes disruptions in circadian rhythms and suppresses the production of melatonin at night, disturbing normal sleep patterns and leading to adverse health outcomes, including depression and stress.^11^ Studies in adult populations have reported that circadian disruption by ALAN was associated with increased stress.^12^ Low-income communities in urban settings tend to also have higher air pollution.^13^ Exposure to nitrogen dioxide has been found to be directly associated with poor performance on neurobehavioral tests^14^ and memory evaluations,^15^ while fine particulate matter has been linked with adolescent delinquent behavior.^16^ In Europe, traffic noise ranks second, behind fine particulate matter, as the environmental risk factor with the greatest health impact.^17^ Studies of noise as an environmental stressor have shown associations with a variety of health outcomes,^18^ including direct deleterious associations with children’s behavioral^19^ and mental^20^ health outcomes.

Potentially offsetting the role of these stressors is green space, which appears to have several health benefits.^21^ Generally, residents in neighborhoods with more greenery have been shown to experience less stress,^22^ as green space serves as a buffer from stressful life events and in turn improves perceived mental health.^23,24^ Adolescents have shown decreased aggression with greater neighborhood green space.^25^

In addressing the environmental factors associated with children’s stress, a joint exposure approach enables a more comprehensive examination of the urban environment and the relative associations and potential synergisms of stressors. As described in cumulative risk frameworks, multiple stressors work together to induce disease and should be considered together.^26^ For example, in a prospective cohort of schoolchildren,^27^ green space was found to be associated with enhanced working memory and a reduction in inattentiveness, but the observed outcomes were partially mediated by elemental carbon air pollution exposure. To our knowledge, few other studies have taken a multiexposure approach in assessing mental health outcomes.

In this study, we examined both the interrelationships of several environmental factors, including ALAN, near-roadway air pollution (NRP), noise, and green space, on self-reported stress in a large cohort of southern California children. The roles of sleep and home and neighborhood stressors, including exposure to smoke, population density, and household income, were also addressed. Southern California is home to 4 of the 10 most populated counties in the US, and, at the time of data collection for this study, Los Angeles-Long Beach-Anaheim was the most densely populated urbanized area in the US,^28^ making it a suitable study area for examining the role of the urban environment on stress.

## Methods

**Figure 1.**
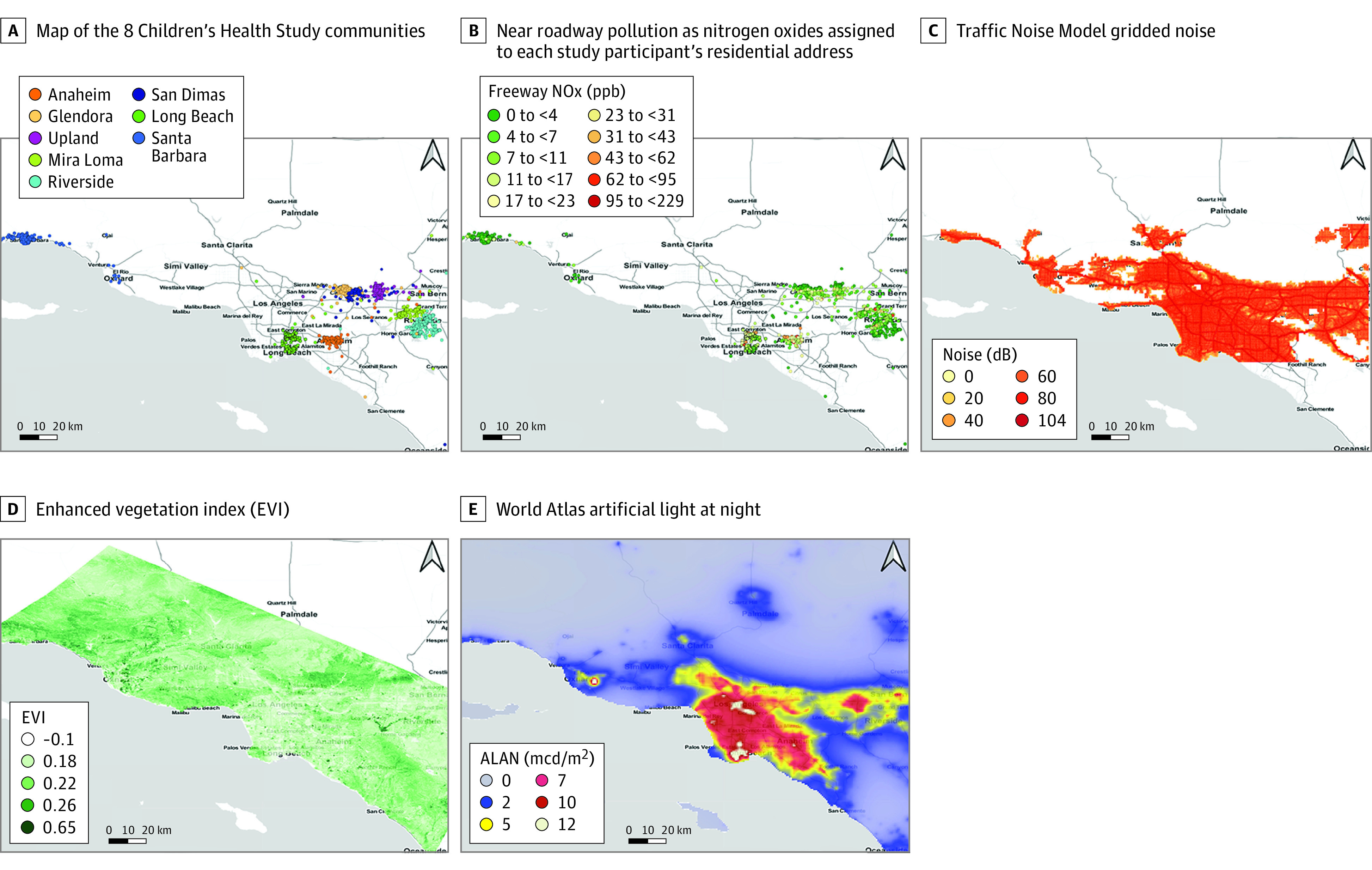
Maps of Study Area and Selected Environmental Exposures A, The 8 Children’s Health Study communities (Anaheim, Glendora, Long Beach, Mira Loma, Riverside, Santa Barbara, San Dimas, and Upland). B, Near roadway pollution as nitrogen oxides (NOx, parts per billion [ppb]) assigned to each study participant’s residential address. C, Traffic Noise Model gridded noise (L_dn_, dB). D, Enhanced vegetation index (EVI). E, World atlas–indicated artificial light at night (ALAN) (millicandela per meter squared [mcd/m^2^]).

 The cohort study population consisted of participants from the Children’s Health Study cohort who were examined in both 2010 and 2012 and resided in 8 urban areas of Southern California (Figure 1A). In each of grades 7 to 8 and 9 to 10, height and weight were measured, and questionnaires covering demographics, home characteristics (including exposure to secondhand smoke), and stress were administered under the supervision of study staff. Children assented to participating in the study and did not receive financial compensation. Study protocols were approved by the institutional review board at the University of Southern California; additional details of this cohort have been described elsewhere.^29^ This study followed the Strengthening the Reporting of Observational Studies in Epidemiology (STROBE) reporting guideline. Three sleep questions, which were part of the 2010 questionnaire, asked about typical duration of sleep (<5 hours to >11 hours in 7 categories), trouble going to sleep (yes, no, sometimes), and trouble staying asleep (yes, no, sometimes). Sleep duration was dichotomized with sleep less than 8 hours considered not enough and 8 hours or more considered enough, and sleep initiation and staying asleep were dichotomized by recoding the response of sometimes to yes. Census block group-level median household income and population density (2010) were linked with each study participant’s geocoded address.

Perceived stress is a self-evaluation of stressful situations in terms of being able to assess and cope with negative events. The primary tool used to quantify perceived stress was the 4-item version of the Perceived Stress Scale (PSS), a self-report questionnaire developed by Cohen et al^30^ that is widely validated and reliable for use in studies of adolescent populations.^31^ The PSS-4 includes the following questions, each on a 5-point Likert scale: In the last month, how often have you felt (1) that you were unable to control the important things in your life, (2) confident about your ability to handle your personal problems, (3) that things were going your way, and (4) difficulties were piling up so high that you could not overcome them? For each of the 2 periods of time examined (ages 13-14 and 15-16 years), a composite stress score was calculated as the sum of the 4 responses (ranging from 0 to 16, with higher scores indicating greater perceived stress).

Annual 2010 and 2012 ambient concentrations of freeway and nonfreeway NRP, which are localized air pollution concentrations from traffic over and above regional concentrations, were estimated at each study participant’s geocoded residential address from the CALINE4 traffic line source dispersion model.^32^ This model used residential locations, roadway geometry, vehicle traffic volume and emission rate by roadway link, and meteorologic conditions, such as windspeed and wind direction, as inputs. The estimated pollutant exposures decay from the roadway maximum within several hundred meters to near background concentrations and explain much of the local-scale spatial variation in annual average ambient nitrogen dioxide levels. In addition, within each community, central site ambient concentrations of fine particulate matter were measured by Federal Reference Method monitors. Annual 2010 and 2012 concentrations were included as community-level indicators of regional pollution.

Noise is typically assessed with acoustic models; in the US, the most recent validated version of the Federal Highway Administration Traffic Noise Model (TNM2.5)^33,34^ uses roads, traffic volumes, pavement type, and mix of vehicle type to output traffic noise as an average day-night sound level: an average noise decibel level over a day with a 10-dB penalty applied from 10:00 pm to 7:00 am. An increase of 10 dB is perceived as being twice as loud,^35^ and a typical urban neighborhood residential area in the US ranges from 55 to 70 db. The TNM2.5 was applied to produce average noise estimates on a 100-m spatial grid over the study period, which we spatially matched to each Children’s Health Study participant’s residential location.^36^

Nighttime radiances observed in the day-night spectral band from the multispectral polar-orbiting Visible Infrared Imaging Radiometer Suite (VIIRS) satellite provide views of ALAN at a 750-m spatial resolution. The developers of this product filter imagery for contamination^37^ and create pixel-based monthly summaries of night light radiance in nanowatts per meter squared (nW/m^2^) that is attributed to artificial sources such as buildings, streetlights, and cars. We extracted monthly VIIRS data in 2012 (the first year of available data from the satellite instrument) and used their average for our analyses. In an effort to capture a more representative human exposure of “skyglow,” the world atlas of artificial night sky brightness represents a total sky brightness as a luminance^38^ and is based on 2015 VIIRS ALAN with the addition of brightness measurements from handheld and vehicle-mounted sky-quality meters. The world atlas of ALAN represents luminance in millicandela per meter squared (mcd/m^2^).

Estimates of greenness were derived from the moderate resolution imaging spectroradiometer satellite instrument. Vegetation-informative spectral bands are combined into metrics representing greenness; the most common of these is the normalized vegetation index, which has been used in several studies examining local green space and health.^22^ The enhanced vegetation index (EVI) incorporates additional spectral information to capture tree canopy structural variations, leaf area index, and canopy architecture.^39,40^ Both the normalized vegetation index and EVI have a theoretical range of values from −1 to 1, where values of 0.1 and below correspond to barren areas, while high values (>0.6) indicate dense vegetation.^41^ Using biweekly 250-m resolution data, we created per-pixel annual averages for 2010 and 2012 and linked them to each study participant’s geocoded residence.

### Statistical Analysis

Analysis was conducted from February 6 to August 24, 2019. Summary statistics, including Spearman correlations, were used to characterize the study population. Two-sample *t* tests were used to compare differences in stress between groups. Our main analyses used mixed-effects regression with a random effect for participants to consider dependencies between repeatedly measured observations within participants and fixed effects for community- and participant-specific covariates. In these models, we examined associations between each exposure of interest separately in single-exposure models and together in multiexposure models. To address potential nonlinearities in the exposure-response associations of continuous variables, we examined nonparametric functions using generalized additive models with adaptive spline smoothers, which facilitated the selection of knot locations. For sections of a particular variable that were near linear, we fit piecewise linear splines at adaptive generalized additive model–selected knot locations, which have the advantage of interpretability of the estimated regression coefficient.

Effect size modification of the environmental factors by population and median household income was examined. We categorized each modifier by the quartiles of their respective distributions and examined interactions with the environmental factors. We also conducted a mediation analysis of sleep (initiation, ability to stay asleep, and duration) because it could be a potential mechanism underlying the association between the environmental factors and stress. Mediation was assessed in a 4-step manner^42^ and is quantified by the proportion of the association between the environmental factor and stress (β_E_) that is explained by sleep (β_EM_),^43^ expressed as a percentage: 100% (1 − β_EM_/β_E_).

The results of all models are expressed as the change in PSS-4 score associated with an interquartile range (IQR) change in exposure. Statistical significance was set at a 2-sided *P* value of 0.05 or less. All analyses were conducted in the R Language, version 3.6.1 (R Foundation for Statistical Computing).

## Results

### Perceived Stress by Group

There were 2290 children with a mean (SD) age of 13.5 (0.6) years who responded to the PSS-4 questions in 2010 (Table 1). Approximately 39% of the participants were lost to follow-up, with 1390 children responding in 2012 at a mean age of 15.3 (0.6) years. The loss to follow-up did not appear to be associated with the outcome; the mean PSS-4 score in 2010 was 5.3 (3.3) and in 2012 was 5.4 (3.3), which was not a statistically significant difference.

**Table 1.** Children’s Health Study Population and Exposure Characteristics

Characteristic	No. (%)	
	2010	2012
Sex	2290	1390
Boys	1141 (50)	688 (49)
Girls	1149 (50)	702 (51)
Age, mean (SD), y	13.5 (0.6)	15.3 (0.6)
Height, mean (SD), cm	159.9 (8.3)	165.7 (8.7)
Weight, mean (SD), kg	55.9 (15.1)	65.3 (16.7)
Exposed to secondhand smoke	189 (8.3)	83 (6.0)
Race/ethnicity		
Asian	108 (5)	73 (5)
African American	56 (2)	45 (3)
White	919 (40)	485 (35)
Mixed	292 (13)	156 (11)
Other	588 (26)	396 (28)
Unknown or missing	327 (14)	235 (17)
Hispanic	1291 (56)	839 (60)
Non-Hispanic	866 (38)	463 (33)
Unknown or missing	133 (6)	88 (6)
Trouble initiating sleep	1239 (54)	NA
Trouble staying asleep	464 (20)	NA
Sleep ≥8 h	1357 (59)	NA
Exposures, mean (SD)		
NRP, ppb^a^		
Freeway	13.2 (16.3)	12.4 (15.0)
Nonfreeway	4.04 (2.83)	4.05 (2.63)
Noise, dB	72.3 (7.84)	73.1 (7.57)
ALAN		
VIIRS, nW/cm^2^/sr^b^	24.0 (15.80)	26.3 (17.27)
World atlas, mcd/m^2^^c^	4.03 (2.16)	4.28 (2.53)
NDVI	0.34 (0.08)	0.32 (0.08)
EVI	0.20 (0.05)	0.19 (0.04)
Communities		
Anaheim	211 (15)	211 (15)
Glendora	69 (5)	69 (5)
Long Beach	178 (13)	178 (13)
Mira Loma	121 (9)	121 (9)
Riverside	239 (17)	239 (17)
Santa Barbara	308 (22)	308 (22)
San Dimas	126 (9)	126 (9)
Upland	138 (10)	138 (10)
PSS-4, mean (SD)^d^	5.28 (3.29)	5.35 (3.30)

Abbreviations: ALAN, artificial light at night; EVI, enhanced vegetation index; mcd/m^2^, millicandela per meter squared; NA, not applicable; NDVI, normalized difference vegetation index; NRP, near-roadway air pollution; nW/m^2^ per centimeter, nanowatts per meter squared; ppb, parts per billion; PSS-4, 4-item Perceived Stress Scale; VIIRS, Visible Infrared Imaging Radiometer Suite.

^a^ Nitrogen oxides.

^b^ Artificial light at night from the Visible Infrared Imaging Radiometer Suite in nanowatts per centimeter squared per steradian.

^c^ Artificial light at night from the world atlas of artificial night light.^38^

^d^ Score scaled from 0 to 16, with higher scores indicating greater perceived stress.

Overall, girls had a significantly higher mean (SD) PSS-4 score (5.7 [3.4]) than boys (4.9 [3.2]). With age, the PSS-4 score increased from 5.6 (3.3) to 6.0 (3.4) in girls but decreased for boys from 5.0 (3.2) to 4.7 (3.1). The PSS-4 score was notably higher for Hispanic than non-Hispanic children (5.5 [3.4] vs 5.0 [3.2]). Other differences in the PSS-4 score were observed in children who were exposed to smoke at home (6.7 [3.1]) vs those who were not exposed (5.4 [3.2]). Weight and body mass index were each significantly associated with increased PSS-4, with body mass index showing a larger increase (0.35; 95% CI, 0.21-0.49) per IQR increase (6.21; calculated as weight in kilograms divided by height in meters squared). The association between stress and height was opposite that of body mass index; per IQR increase in height (30.5 cm), there was a statistically significant decrease in stress (−0.35; 95% CI, −0.50 to −0.20). Given these associations, all subsequent models were adjusted for sex, race/ethnicity, exposure to secondhand smoke, body mass index, and height.

### Environmental Exposures

Maps (Figure 1) and boxplots (eFigure in the Supplement) show the study region and distribution of select factors of the built environment. We observed NRP to be highest for children in Anaheim (mean [SD], 34.8 [26.5] ppb), as was traffic noise (77.4 [6.8] dB), which was predictably higher on freeways and major roads. Greenspace (EVI) was higher in Santa Barbara (mean [SD], 0.23 [0.05]) and Glendora (mean [SD], 0.22 [0.03]), while world atlas ALAN peaked in Long Beach (7.85 [0.63] mcd/m^2^) near the ports and was also high in Anaheim (7.85 [0.68] mcd/m^2^). Spearman correlations between environmental factors (eTable 1 in the Supplement) indicated that ALAN was moderately negatively correlated with the greenness indices (Spearman r = −0.72) and moderately correlated with NRP (Spearman r = 0.58 for freeway and Spearman r = 0.70 for nonfreeway).

### Main Analyses

Single-exposure models (Table 2) report that the sum of freeway and nonfreeway NRP (ie, total NRP) has a stronger association with stress than either of its components. An IQR increase in total NRP resulted in a 0.17 (95% CI, 0.05-0.28) increase in the PSS-4 score. World atlas ALAN showed the greatest association with stress, in which an IQR increase was associated with a 0.44 (95% CI, 0.03-0.84) increase in the PSS-4 score. The EVI had a slightly greater protective effect and a narrower 95% CI (−0.29; 95% CI, −0.47 to −0.10) than the normalized vegetation index.

**Table 2.** Estimated Linear Associations Between Stress and Environmental Factors From Single-Exposure Models

Environmental factor	Environmental factor IQR	Effect estimate (95% CI)^a^	*P* value
Exposure to secondhand smoke	NA	1.23 (0.84-1.62)	<.001
ALAN, VIIRS, nW/cm^2^/sr^b^	2.44 (12.2 to 35.2)	0.23 (0.02 to 0.45)	.03
ALAN, world atlas, mcd/m^2^	2.44 (2.53 to 4.98)	0.44 (0.03 to 0.84)	.04
EVI	0.06 (0.17 to 0.23)	−0.29 (−0.47 to −0.10)	.003
NDVI	0.11 (0.27 to 0.38)	−0.28 (−0.50 to −0.07)	.008
NRP, ppb^c^			
Total	13.2 (7.49 to 20.73)	0.17 (0.05 to 0.28)	.005
Freeway	11.1 (4.39 to 15.52)	0.14 (0.04 to 0.24)	.005
Nonfreeway	3.34 (2.05 to 5.39)	0.05 (−0.15 to 0.25)	.10
Noise, dB	10.2 (67.38 to 77.61)	0.10 (−0.06 to 0.26)	.63

Abbreviations: ALAN, artificial light at night; EVI, enhanced vegetation index; IQR, interquartile range; mcd/m^2^, millicandela per meter squared; NA, not applicable; NDVI, normalized difference vegetation index; NRP, near-roadway pollution; nW/m^2^ per centimeter, nanowatts per meter squared; ppb, parts per billion; VIIRS, Visible Infrared Imaging Radiometer Suite.

^a^ All models adjusted for height, body mass index, race, ethnicity, community, and effect estimates of continuous variables are scaled by their IQR.

^b^ Artificial light at night from the Visible Infrared Imaging Radiometer Suite in nanowatts per centimeter squared per steradian.

^c^ Nitrogen oxides.

For similar environmental factors (normalized vegetation index, EVI and ALAN VIIRS, and ALAN world atlas), we chose the factor that was more associated with stress from the single-exposure models. Although noise was not significantly associated with stress in the single-exposure model (Table 2), we examined whether an association was noted in the multiexposure model as it has previously been suggested to be an important determinant of stress in children.^35^

**Figure 2.**
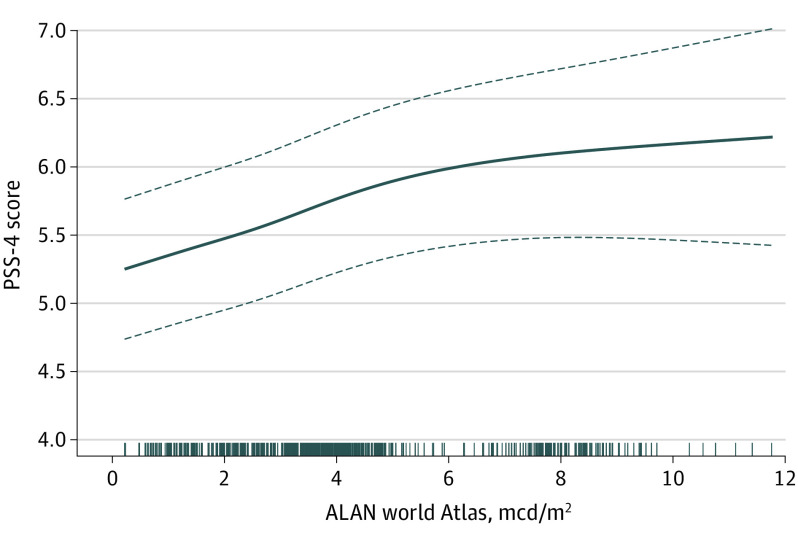
Nonlinear Association Between World Atlas–Indicated Artificial Light at Night (ALAN) and Perceived Stress Scale (PSS-4) in the Multi-Exposure Model Solid line represents the mean regression curve, and dashed lines are the 95% CIs. mcd/m^2^ indicates millicandela per meter squared.

The world atlas ALAN showed a nonlinear association in the multiexposure model, but all of the other environmental factors remained linear. Adaptive smoothing splines of ALAN estimated knot placement at 4.9 and 7.1 mcd/m^2^ (Figure 2). Fitting a piecewise linear spline with these knots, we found a statistically significant increase in the PSS-4 score per IQR increase in ALAN between 0 and 4.9 mcd/m^2^ (0.57; 95% CI, 0.05-1.09) (Table 3). The associations with the other 2 pieces of the spline, ie, between 4.9 to 7.1 mcd/m^2^ and greater than 7.1 mcd/m^2^, were not statistically significantly different than 0. Per IQR increase in the respective exposures, the effect estimate for NRP (0.16; 95% CI, 0.02-0.30) was only slightly attenuated in the multiexposure model, as was EVI (−0.24; 95% CI, −0.45 to −0.04), which partially mitigated the detrimental impact of the stressors. Traffic-related noise remained nonsignificant, but we found that exposure to secondhand smoke was associated with the greatest increase in the PSS-4 score (0.85; 95% CI, 0.46-1.24). Models including community-specific central site fine particulate matter monitor concentrations together with deviations of NRP from its community mean suggested a minimal and nonsignificant impact of regional pollution (eTable 2 in the Supplement).

**Table 3.** Estimated Associations Between Stress and Environmental Factors From the Multiexposure Model

Environmental factor	Effect estimate (95% CI)^a^
Exposure to secondhand smoke	0.85 (0.46-1.24)^b^
ALAN, world atlas (piecewise linear), 0-4.9 mcd/m^2^	0.57 (0.05-1.09)^b^
Total NRP, ppb^c^	0.16 (0.02-0.30)^b^
EVI	−0.24 (−0.45 to −0.04)^b^
Noise, dB	−0.12 (−0.32 to 0.08)

Abbreviations: ALAN, artificial light at night; EVI, enhanced vegetation index; mcd/m^2^, millicandela per meter squared; NRP, near-roadway air pollution; ppb, parts per billion.

^a^ All models adjusted for race, ethnicity, body mass index, height, community, and other environmental factors; effect estimates are scaled by their interquartile range.

^b^ *P* < .05.

^c^ Nitrogen oxides.

### Effect Modification and Mediation

When examined individually as interactions in the multiexposure model, only median annual household income was a statistically significant modifier. Community-specific distributions (eFigure in the Supplement) showed the highest values in the communities of Glendora (median household income: $100 875; IQR, $71 638-$107 727) and Upland ($96 358; IQR, $82 750-$111 016). Lowest household incomes were in Long Beach ($48 750; IQR, $38 167-$67 239) and Mira Loma ($50 362; IQR, $44 716-$73 611). Summarized over all communities, the lowest quartile of income was $48 153. As an effect modifier, we found that study participants below the 25th percentile of median household income had significantly higher stress per IQR increase in ALAN (0.71; 95% CI, 0.11-1.30).

Sleep initiation (*t* value = −9.05, *P* < .001), staying asleep (*t* value = −11.3, *P* < .001), and sleep duration (*t* value = −3.80, *P* < .001) were each associated with stress; however, only sleep duration was statistically significantly associated with any of the environmental factors. We observed a 32% decrease (*P* = .002) in getting at least 8 hours of sleep per IQR increase in world atlas ALAN and a 28% increase (*P* = .03) in getting at least 8 hours of sleep per IQR increase in the EVI. In the multiexposure model, including sleep duration reduced the magnitude of the ALAN and EVI associations, but they remained statistically significant. Sleep mediated 18% of the association between ALAN and stress and 17% of the association between EVI and stress (eTable 3 in the Supplement).

## Discussion

We studied the association of multiple urban environmental exposures with self-reported perceived stress in Southern California children aged 13 to 16 years. To our knowledge, this is one of the first studies to take a multiexposure approach to examining stress in children of these age groups. Findings on the study population included the following: girls reported higher stress levels than boys, particularly later in adolescence; Hispanic children reported more stress than their non-Hispanic classmates; and exposure to smoke in the home was considered a substantial factor in perceived stress. The association between poor mental health and secondhand smoke has been examined previously^44^ and was associated with increased odds of depression in Korean adolescents,^45^ self-reported psychological distress in Spanish adolescents,^46^ and self-reported antisocial dispositions in Québec children.^47^ While the biological and physical mechanisms remain poorly understood, secondhand smoke is often considered to be a proxy for stressful living conditions.^44^

Among the physical environmental exposures, ALAN showed the largest association with increased perceived stress. We observed that the association was nonlinear, in which the primary dose-response signal occurred between 0 and 5 mcd/m^2^. The developers of the world atlas^38^ indicate that locations with skyglow above 1.4 mcd/m^2^ never experience conditions resembling a true night because it is masked by artificial light. Among the communities studied, only Santa Barbara experiences a mean ALAN below this value.

The mechanism by which ALAN acts on stress is likely through the hypothalamus-pituitary-adrenal axis, which is important to the physiologic regulatory system.^12^ It is well established that ALAN disrupts circadian rhythms and suppresses the production of melatonin, which in turn affects sleep and health.^11,48^ We identified evidence that appears to support this finding, whereby an IQR increase in ALAN was associated with a 32% decrease in getting at least 8 hours of sleep, and it partially explained the association between perceived stress and ALAN.

Earlier versions of coarse resolution satellite-derived ALAN have been used to capture greater total evening and nighttime light generated from street and building lighting as a metric of exposure through commuting, outdoor evening activities, and leakage into the bedroom at night.^49,50^ Previous epidemiologic studies of the health effects of residential outdoor ALAN have primarily examined adults^48^ and have found increases in depression^11^ as well as increased breast^51,52^ and prostate^53^ cancer risk.

A recent study suggested that, while satellite-assessed outdoor ALAN exposure levels are correlated with urban environmental exposures, they may not be a good proxy for indoor evening or nighttime personal exposure.^54^ The investigators found only weak correlations with nighttime bedroom light but, similar to our study, observed a moderate to strong correlation of outdoor ALAN with ambient air pollution. We also observed that traffic-related air pollution is statistically significantly associated with increased stress, albeit with an effect estimate that is 72% less that of ALAN. One possibility is that ALAN may be a better proxy of localized air pollution and better captures other aspects of the built environment, such as well-lit local streets, than air pollution models or population density.

Counterbalancing the combined ALAN and air pollution stressors is green space, which we found to have a moderate protective association with perceived stress. Several studies corroborate our findings, suggesting the benefits of increased green space on both child^55,56^ and adult^57^ mental health. When examined together with traffic-related air pollution, it has been suggested that more neighborhood green space is linked with reduced air pollution exposure,^58^ which in turn was reported to be associated with improvements in cognitive development in schoolchildren.^27^ Increased green space may also show health benefits, as it is linked with reductions in environmental noise.^59^ While increased traffic- and aircraft-related noise have been associated with children’s cognitive outcomes,^60^ we did not observe significant associations between traffic-related noise and stress in any of our models. It is possible that the noise estimates from the model do not adequately reflect perceived noise exposures or capture the direct effect of noise exposures on stress.

This study has several implications for clinicians, policy makers, and urban planners. According to the United Nations, more than 80% of the population of the US lives in urban areas, and urban areas are projected to increase between 2018 and 2050.^61^ Urban areas are complex socioeconomically as well as environmentally, in which the built environment is associated with increased pollution and decreased green space. As suggested in this and other studies, children’s exposure to the urban pollutant mixture may result in declines in mental health. A deeper understanding of a child’s neighborhood can provide insight into how to better characterize health risks and provide targeted clinical care.^62^ Thus, it is important that city planners prioritize the development of or transition to more green spaces in residential areas. For example, the greening of vacant lands, particularly in resource-limited urban settings, has been shown to be a useful treatment for self-reported feelings of depression.^57^ In turn, more green space will lead to reductions in ALAN and traffic-related pollution, which have both been suggested to have significant detrimental associations with stress. Such a change is possible and has the potential of wide-reaching benefits to the health and well-being of both children and adults.

### Limitations

This study has limitations. We used the PSS-4 questions to measure our health outcome: perceived stress. Although the PSS-4 is a validated scale, it is the shortened version of the full set of questions that comprise the 10- and 14-item forms. Thus, there may be less reliability in reported stress than there would have been if the full set of questions were used. A related limitation is response bias that is often found in studies relying on questionnaires for gathering participant-level data. One such concern in this study is nonresponse bias that may occur if children who participated in the initial phase of the study did not respond to the PSS-4 questions in follow-up. As a sensitivity check, analysis conducted only on children who responded to the PSS-4 questions both times yielded the same conclusions for our full multiexposure models. Another limitation is the time matching of environmental exposures with the outcome data, which were not always aligned. While we were able to match 2010 and 2012 NRP and EVI data with the respective years of PSS-4 responses, the ALAN data were available only for 2015, and the noise exposure data from TNM2.5 were available only as an average over 2012-2015. This temporal mismatch may have led to some exposure misclassification in our findings. In addition, we cannot rule out the possibility of unmeasured or residual confounding by other factors that relate to perceived stress, such as neighborhood crime rates or family psychosocial factors.

## Conclusions

In this cohort study, the confluence of ALAN, NRP, noise, and a lack of green space in neighborhoods of Southern California were found to be significantly associated with increased self-reported psychosocial stress in children. Advocating for increased green space and reduced traffic could serve as useful interventions that may have lasting improvements on child mental health.

## Notes

Supplement. **eTable 1.** Spearman Correlations Between Environmental Factors at Residences of Study Participants **eTable 2.** Estimated Associations Between PSS-4 and Environmental Factors From the Multipollutant Model Including Regional PM_2.5_ **eTable 3.** Estimated Associations Between Stress and Environmental Factors From the Multi-Exposure Model Including Sleep Duration **eFigure.** Boxplots of Environmental Factors, Outcome and Modifier by Community
