# Supplementary material for: Association of the Built Environment With Childhood Psychosocial Stress
Source: JAMA Netw Open. 2020 Oct 21;3(10):e2017634. doi: 10.1001/jamanetworkopen.2020.17634 (PMC7578768; doi:10.1001/jamanetworkopen.2020.17634)
Supplement: Supplement. — eTable 1. Spearman Correlations Between Environmental Factors at Residences of Study Participants eTable 2. Estimated Associations Between PSS-4 and Environmental Factors From the Multipollutant Model Including Regional PM2.5 eTable 3. Estimated Associations Between Stress and Environmental Factors From the Multi-Exposure Model Including Sleep Duration eFigure. Boxplots of Environmental Factors, Outcome and Modifier by Community [file jamanetwopen-e2017634-s001.pdf]

Supplementary Online Content

Franklin M, Yin X, McConnell R, Fruin S. Association of the built environment with childhood psychosocial stress. *JAMA Netw Open*. 2020;3(10):e2017634. doi:10.1001/jamanetworkopen.2020.17634

eTable 1. Spearman Correlations Between Environmental Factors at Residences of Study Participants

eTable 2. Estimated Associations Between PSS-4 and Environmental Factors From the Multipollutant Model Including Regional $PM_{2.5}$

eTable 3. Estimated Associations Between Stress and Environmental Factors From the Multi-Exposure Model Including Sleep Duration

eFigure. Boxplots of Environmental Factors, Outcome and Modifier by Community

This supplementary material has been provided by the authors to give readers additional information about their work.

© 2020 Franklin M et al. *JAMA Network Open.*

eTable 1. Spearman correlations between environmental factors at residences of study participants.

	Freeway NRP	Non-Freeway NRP	Total NRP	Regional $PM_{2.5}$	Noise	NDVI	EVI	ALAN VIIRS	ALAN World Atlas
Freeway NRP[a]	1	0.44	0.96	0.19	0.60	-0.52	-0.53	0.58	0.59
Non-Freeway NRP[a]		1	0.65	-0.03	0.45	-0.46	-0.52	0.70	0.57
Total NRP[a]			1	0.15	0.66	-0.57	-0.59	0.68	0.64
Regional $PM_{2.5}$[b]				1	-0.16	-0.31	-0.11	0.01	0.40
Noise					1	-0.29	-0.39	0.49	0.21
NDVI[c]						1	0.95	-0.66	-0.66
EVI[d]							1	-0.72	-0.72
ALAN VIIRS[e]								1	0.71
ALAN World Atlas[f]									1

[a] near-roadway pollution, nitrogen oxides (NOx); [b] particulate matter with aerodynamic diameter < 2.5um; [c] Normalized Difference Vegetation Index; [d] Enhanced Vegetation Index; [e] Artificial light at night from the Visible Infrared Imaging Radiometer Suite; [f] Artificial light at night from the World Atlas of Artificial Night Light

© 2020 Franklin M et al. *JAMA Network Open.*

eTable 2. Estimated associations between PSS-4 and environmental factors from the multipollutant model including regional $PM_{2.5}$

Environmental Factor	Effect Estimate (95% CI)[a]
Exposure to Secondhand Smoke	0.85 (0.46, 1.24) [b]
ALANc, World Atlas (piecewise linear) (0 – 3.3 mcd/m^2)	0.78 (0.04, 1.53) [b]
Total NRP[d] deviation from mean (ppb)	0.11 (0.01, 0.21)[b]
Regional $PM_{2.5}$ [e](ug/m^3)	0.06 (-0.16, 0.28)
EVI[f]	-0.24 (-0.45, -0.04)[b]
Noise (dB)	-0.12 (-0.31, 0.08)

[a] All models adjusted for race, ethnicity, body mass index, height, community; effect estimates are scaled by the IQR; [b] p<0.05; [c] Artificial light at night; [d] near-roadway pollution; [e] particulate matter with aerodynamic diameter < 2.5um; [f] Enhanced Vegetation Index

© 2020 Franklin M et al. *JAMA Network Open.*

eTable 3. Estimated associations between stress and environmental factors from the multi-exposure model including sleep duration

Environmental Factor	Effect Estimate (95% CI)[a]
Exposure to Secondhand Smoke	0.79 (0.40, 1.18) [a]
ALAN[c], World Atlas (piecewise linear) (0 – 4.9 mcd/m^2)	0.47 (0.00, 0.99) [a]
Total NRP[d] (ppb)	0.16 (0.03, 0.30)[a]
EVI[e]	-0.20 (-0.40, 0.00)[a]
Noise (db)	-0.10 (-0.30, 0.09)
Sleep Duration (>8hrs)	-0.99 (-1.23, -0.75) [a]

[a] All models adjusted for race, ethnicity, body mass index, height, community; effect estimates are scaled by the IQR; [b] $p<0.05$; [c] Artificial light at night; [d] near-roadway pollution; [e] Enhanced Vegetation Index

© 2020 Franklin M et al. *JAMA Network Open.*

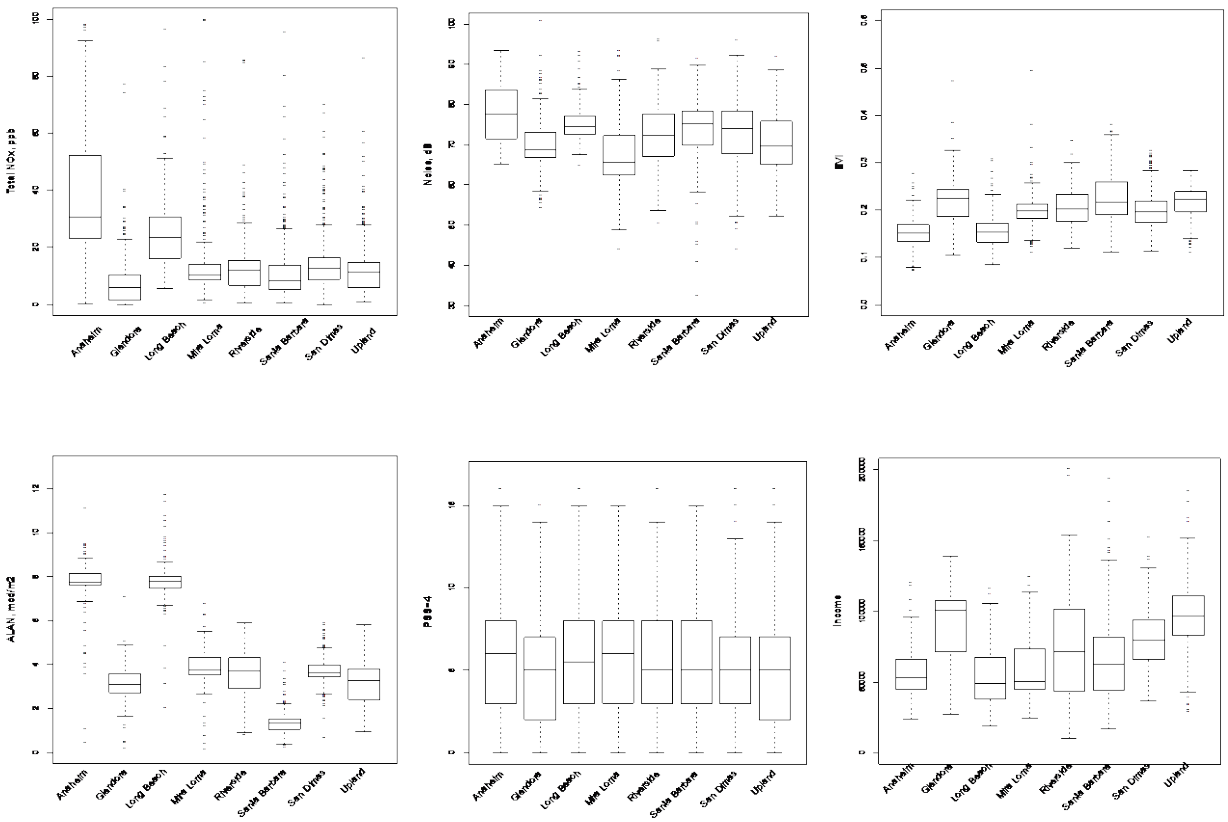

eFigure. Boxplots of environmental factors, outcome and modifier by community (Top left to bottom right: near-roadway air pollution (NOx, ppb), noise (L_{dn}, dB), enhanced vegetation index (EVI), artificial light at night (ALAN) from World Atlas (mcd/m^2), perceived stress (PSS-4), Median Household Income.

© 2020 Franklin M et al. *JAMA Network Open.*
